# Reply to Araújo: Good science requires focus

**DOI:** 10.1073/pnas.2420476121

**Published:** 2024-12-11

**Authors:** Sarab S. Sethi, Avery Bick, Ming-Yuan Chen, Renato Crouzeilles, Ben V. Hillier, Jenna Lawson, Chia-Yun Lee, Shih-Hao Liu, Celso Henrique de Freitas Parruco, Carolyn M. Rosten, Marius Somveille, Mao-Ning Tuanmu, Cristina Banks-Leite

**Affiliations:** ^a^Department of Life Sciences, Imperial College London, London W12 0BZ, United Kingdom; ^b^Norwegian Institute for Nature Research, Trondheim 7034, Norway; ^c^Department of Forestry and Resources Conservation, National Taiwan University, Taipei 106, Taiwan; ^d^Mombak, São Paulo 04547-000, Brazil; ^e^Division of Biosciences, University College London, London, United Kingdom; ^f^Biodiversity Research Center, Academia Sinica, Taipei 11529, Taiwan; ^g^Independent Researcher, Brazil

In their reply to our article, “*Large-scale avian vocalization detection delivers reliable global biodiversity insights*” (1), Araújo (2) raises concerns about our detections of neotropical “birdcalls” [in reality, BirdNET detects both songs and calls (3)]. Although there is certainly value in further investigating the performance of bird vocalization detection models across many different contexts—including the Neotropics—the comment contains some inconsistencies and misunderstands the core positioning and novelty of our work.

First, our study did not claim to perform a comprehensive survey of bird communities in any of the four locations. We acknowledged throughout the paper that discrepancies exist between total expected community sizes and the numbers of species detected by BirdNET, and therefore, we focussed on species trends and dynamics rather than community-level analyses (e.g., measures of species richness). Araújo suggests we “should detect” 56 species per recorded day of audio in our Brazilian dataset. Besides the fact that this number is based on a habitat mismatch, the comment misses the key novelty of our study: To systematically measure and validate what BirdNET was able to detect in each location, rather than making any claims relating to species that were missed.

Second, our study did not train and evaluate new vocalization detection models and, therefore, is not well-positioned to provide detailed recommendations such as the inclusion of specific sets of new training data into BirdNET. Vocalization detection models are data hungry (4), and although WikiAves and Fonoteca Neotropical Jacques Vielliard contain 360,000 labeled recordings, Xeno-Canto and the Macaulay Library together host well over 90 million. Additionally, even regional datasets suffer from taxonomic and geographic biases and long-tailed distributions. For example, of the 1,962 species in WikiAves, only 1.7% (34 species) have over 1,000 samples (5). Integrating smaller regional datasets *might* improve model performance, but without performing controlled computational experiments in which multiple candidate models are compared, publishing recommendations of this sort would not be appropriate.

Third, our study did not compare different approaches for acoustic monitoring of avian species. Most of Araújo’s comments and statistics are from their own impressive work on acoustic surveys of neotropical birds (6). However, to analyze data in their study, experts manually annotated thousands of audio files, and no automated analyses were used, limiting the validity of any comparisons. There are several other ways to derive avian insight from acoustic data including semiautomated annotation (7), soundscape methods (8), or fully unsupervised approaches (9). Comparing derived species and community data across each of these methods is an active area of research (10) but is beyond the scope of our study.

We agree that more research exploring the above three themes would have value. Although this was never the intended purpose (nor claimed impact) of our work, we wholeheartedly encourage others to take this forward. Scientific challenges are multifaceted but still require focus and cannot all be solved in a single step without significantly compromising rigor or quality.
